# Health Literacy is Essential to ASCVD Prevention in Youth

**DOI:** 10.1007/s11883-023-01086-2

**Published:** 2023-02-09

**Authors:** Harnoor K. Mann, Jared W. Magnani, Amber E. Johnson

**Affiliations:** 1 UPMC Department of Internal Medicine, Pittsburgh, PA USA; 2 Center for Research On Health Care, Department of Medicine, University of Pittsburgh, Pittsburgh, PA USA; 3 Department of Medicine, University of Pittsburgh, School of Medicine, 200 Lothrop St, Presbyterian South Tower, Third Floor, WE363.2, Pittsburgh, PA 15213 USA

**Keywords:** Primordial prevention, Primary prevention, Atherosclerosis, Cardiovascular disease, Health literacy, Youth

## Abstract

**Purpose of Review:**

Health literacy is fundamental to primary and primordial prevention of atherosclerotic vascular disease (ASCVD) in children and adolescents. Here we summarize essential components of interventions which address health literacy challenges to reduce ASCVD risk in youth.

**Recent Findings:**

There is a global pandemic of suboptimal health behaviors among youth that may contribute to the increasing rates of ASCVD worldwide. Deficiencies in youth cardiovascular health have promoted increased attention to health education that incorporates health literacy. Studies conducted in both the child (0 to 9 years) and adolescent (10 to 17 years) population have shown improvement in health knowledge, health behaviors such as physical activity and eating habits, and objective measures such as body mass index (BMI), blood pressure, and serum lipid levels. The available literature affirms that the involvement of family and community members in young people’s surroundings—including parents, teachers, and peers—can influence educational interventions’ protective effects.

**Summary:**

Educational interventions which incorporate health literacy have demonstrated potential to address ASCVD risk factors in youth and may be augmented by caregiver and community involvement.

## Introduction

Atherosclerosis is a lifelong disease, with vascular remodeling occurring throughout the lifespan [1–4]. Individuals with cardiovascular risk factors have 6 to 12 times greater likelihood of having or being diagnosed with atherosclerotic cardiovascular disease (ASCVD) in their lifetime [4]. Commonly recognized ASCVD risk factors include obesity, smoking, poor diet, low physical activity levels, inadequate sleep, elevated cholesterol, hypertension, and elevated blood glucose [5, 6]. The pathogenesis of atherosclerosis may even initiate in utero, as exposure to maternal pre-eclampsia is associated with ASCVD in offspring [7, 8]. Postmortem data show over 50% of youth by the ages 2 to 15 years have evidence of early ASCVD [2, 9]. Given the prevalence of ASCVD at an early age, prevention efforts in youth are paramount.

The presence and severity of ASCVD risk factors may be exacerbated by an individual’s social context, including level of education and health literacy [10–12]. Health literacy, as defined by the World Health Organization, is the “degree to which individuals have the capacity to obtain, process, and understand basic health information and services needed to make appropriate health decisions.” [11] Therefore, knowledge-based or behavior modification interventions designed to prevent the development of ASCVD must be implemented in ways that are feasible and appropriate for an individual’s social context [10, 13]. Data suggest that the implementation of accessible educational interventions that are appropriate to the participants’ level of health literacy can effectively reduce ASCVD risk and its development. In this review, we assess the central role of health literacy in educational and behavioral modification interventions targeting primordial and primary prevention in youth.

## Health Literacy as a Social Determinant of Health

Social deprivation contributes to ASCVD risk in youth and towards limited opportunities for health-related education and health literacy. Youth health literacy is shaped by educational access, financial limitations, and other social determinants of health (SDOH). Data from China demonstrate decreased number of ideal cardiovascular health factors in children from families of lower education and lower income levels [14]. In a cross-sectional study conducted in a European cohort of 2100 adolescents, those that pursued apprenticeships rather than vocational or general academic high schools had poorer diets (43.6% vs. 35.7% vs 26.6%; *p* < 0.001) and were more likely to have BMI > 95th percentile (16.2% vs. 8.9% and 4.2%; *p* < 0.001) [5, 15]. Adverse childhood experiences such as physical or psychological abuse, caregiver substance use disorders, and intimate partner violence in the home are additionally associated with suboptimal health behaviors, thereby exacerbating ASCVD risk [16•, 17]. Children with 4 or greater adverse childhood experiences, in comparison to those with none, are twice as likely to smoke (OR 2.2; CI, 1.7–2.9), 1.6 times more likely to have a BMI ≥ 35 kg/m^2^ (OR 1.6; CI, 1.2–2.1), and twice as likely to be diagnosed with ischemic heart disease (OR 2.2; CI, 1.3–3.7) in adulthood [17]. Children from families with lower incomes are also more likely to experience an increased number of adverse childhood experiences than children from families with middle or higher incomes [18]. Associations between social determinants of health and ASCVD risk factors further emphasize the need for risk mitigation in youth through targeted, educationally appropriate programming.

## Health Literacy Affects Youths’ Health Knowledge and Behaviors

Knowledge and awareness of ASCVD are fundamentally limited in youth. In a survey of 331 young women 15 to 24 years old, only 10% correctly identified heart disease as the leading cause of death for women [19]. A qualitative study conducted in the UK found that although most adolescents had heard of cardiovascular disease, and all knew the risk of disease was augmented by unhealthy lifestyle practices, some thought that lifestyle changes did not need to occur until they were older as participants perceived ASCVD as a disease acquired in middle age [20]. Participants also had difficulty specifically describing how lifestyle behaviors were associated with ASCVD. Integral gaps in ASCVD knowledge, the prominence of risk factors, and suboptimal health behaviors emphasize the need for educational interventions in youth.

Deficiencies in youth health behaviors and objective health factors have been well-established globally [14, 15, 21, 22]. In an analysis of data from the National Health and Nutrition Examination Survey from 2005 to 2010, none of 4673 participants ages 12 to 19 reported having an ideal Healthy Diet Score [23]. Additionally, more than one-third had poor smoking status and a BMI that did not fit the ideal benchmark. Such rates of cardiovascular health behaviors are similar to data obtained from Chinese, European, and Caribbean cohorts [14, 15, 21, 22]. The presence of ASCVD risk factors in youth has been predictive of physiologic markers of ASCVD [24]. An analysis of data from 387 healthy Italian adolescents showed inverse associations between cardiovascular health scores and carotid-femoral pulse wave velocity, a validated marker of atherosclerosis [24].

## Interventions Considering Health Literacy in Children

Beginning in 2009, an international group of three cluster-randomized controlled trials ranging in size from 500 to 2000 children was sequentially conducted using a structured preschool-based educational intervention designed to reduce ASCVD development [25–27, 28••]. The trials were conducted in Bogotá, Colombia, Madrid, Spain, and New York, New York. All three trials assessed the effect of a program focused on changing knowledge, attitudes, and habits towards healthy nutrition and physical activity. The intervention consisted of 30–50 h of structured curricula focused on physiology, nutrition, and physical activity. Each intervention was designed for participants’ current level of level of education. Activities included age-appropriate stories, videos, games, and songs [29]. Parents and families also attended school-wide events and were encouraged to participate in take-home exercises. Preschools in Bogotá were chosen specifically prior to randomization to ensure representation of migrant Colombian unprivileged communities [26]. The Spanish trial was designed using stratified randomization based to accommodate for varied immigrant backgrounds and household incomes at each of the preschools [30]. The American trialists chose to conduct the intervention in the Harlem neighborhood of New York, an underserved, urban, ethnically, and racially diverse community. Hence, the investigators assessed the intervention in socially diverse preschools where students encountered additional challenges of social determinants of health.

Knowledge, attitude, and habits were measured using standardized instruments administered by trained professionals. Questionnaire items included “location of the heart,” “recognizes a variety of possibilities for carrying out physical activity,” identifying inappropriate nutrition “once in a while foods,” and quantifying recent type of food consumption and physical activity [26]. In all three trials, children in the intervention groups demonstrated statistically significant increases in knowledge, attitude, or habit sub-scores immediately post-intervention. Analyses conducted in the Colombian cohort at 3 years post-intervention showed improvements in composite knowledge, attitude, and habit scores. The American cohort showed a dose–response effect of the educational intervention, as children who participated in > 75% of modules had a larger change in scores than those who participated in 50–75%, and those with < 50% completion showed the least improvement. The Spanish and American cohorts noted an increase in composite knowledge, attitude, and habit scores in children from families from higher educational backgrounds and higher incomes, reflective of an association between socioeconomic status and ideal health [28••, 30]. In the Colombian cohort, at the 3-year timepoint, 540 children underwent additional physiologic measurements. BMI ranges were defined using Centers for Disease Control and Prevention growth charts. Sixty-two children went from the underweight to normal weight category, and 91 went from being overweight/obese to normal weight category. The Spanish and American cohorts did not show such changes in BMI. All three trials demonstrated meaningful and sustained improvements in awareness of ASCVD and risk factor modification.

Another educational trial conducted in Brazil among older children showed similar results [31]. The educational intervention, tailored towards children 7–11 years old, consisted of an 8-week after-school workshop series with sessions lasting 30 to 90 min each. The workshops included art activities, games, music, dance, and real-life simulations that all centered around healthy habits and cardiovascular health at an appropriate literacy level. Baseline questionnaires assessed cardiovascular knowledge, and participant knowledge was reassessed after completion of the intervention. The 79 participants were all enrolled from an after-school program for children of lower income backgrounds. The results of this study showed that children in the intervention group had a 1.4-point increase in knowledge of health habits immediately post-intervention on a 5-point scale, which was sustained at a 1-point increase at 3 months (1.4, CI 0.9–2.0 vs 1.0, CI 0.3–1.6; *p* < 0.001). This trial affirms the use of educational programming in children in an older age range.

## Interventions Considering Health Literacy in Adolescents

Educational interventions in adolescents demonstrate similar shifts in ASCVD risk factor management as those in children. Project Healthy Schools, an initiative from the University of Michigan and local community organizations, is a school-based program designed to promote healthier choices to reduce youth obesity and long-term ASCVD risk [32, 33]. The 10-week program focuses on diet and physical activity behavior change through ten 20-min interactive educational modules during the school day. Prospective studies of the intervention recorded outcomes of health behavior change after program completion, including dietary habits, physical activity, sedentary activity, and physiological outcomes including serum lipid levels, blood pressure, random blood glucose, and BMI.

Separately, two prospective studies of 1100–4000 middle school students in Michigan showed post-participation improvement in physical activity, with the larger study showing increases in participation in both moderate (3.16 to 3.54 days per week; *p* < 0.001) and vigorous (4.13 to 4.52 days per week; *p* < 0.001) physical activity [32]. In the larger of the two studies, significant dietary improvement defined as improved fruit and vegetable intake was shown as well. Among objective measures, improvement in serum cholesterol markers were noted in both studies. The larger study also found significant decreases in random blood glucose (97.51 ± 16.00 to 94.94 ± 16.62; *p* < 0.001), systolic blood pressure (109.47 ± 15.26 mm Hg to 107.76 ± 10.87 mm Hg; *p* < 0.001), and diastolic blood pressure (64.78 ± 8.57 mm Hg to 63.35 ± 7.81 mm Hg; *p* < 0.001) [32]. The median income of families whose children participated in the larger study ranged from $28,610 to $56,612. Project Healthy Schools, in successfully teaching students from economically limited backgrounds, reaffirms the importance of accessible and grade-relevant programming in ASCVD prevention.

Similar to Project Healthy Schools, the “Wizards of Motion Cardiovascular Disease Prevention Module” focused on ASCVD and its prevention, but did so through experimental learning [34]. The educational module was designed to introduce basic cardiac physiology to 5th grade students to help them identify relationships between the cardiovascular system and health habits, such as healthy food intake, smoking avoidance, and regular physical exercise. Students were randomized to either the intervention group that received the educational module or a control group that received standard school curriculum. After an age-appropriate educational presentation, students participated in a model-based laboratory activity designed to simulate restriction of coronary artery flow using rubber tubing, flow valves, clamps, and colored water. Assessment of knowledge and attitudes towards ASCVD prevention followed the educational intervention. Students who participated in the module had a greater than 60% increase in knowledge test (28.21% vs 94.87%; *p* < 0.01) as well as greater than 10% increase in the attitude test score (72.67% vs 83.15%; *p* < 0.01) [34].

## Need for Family and Community Integration

**Fig. 1:**
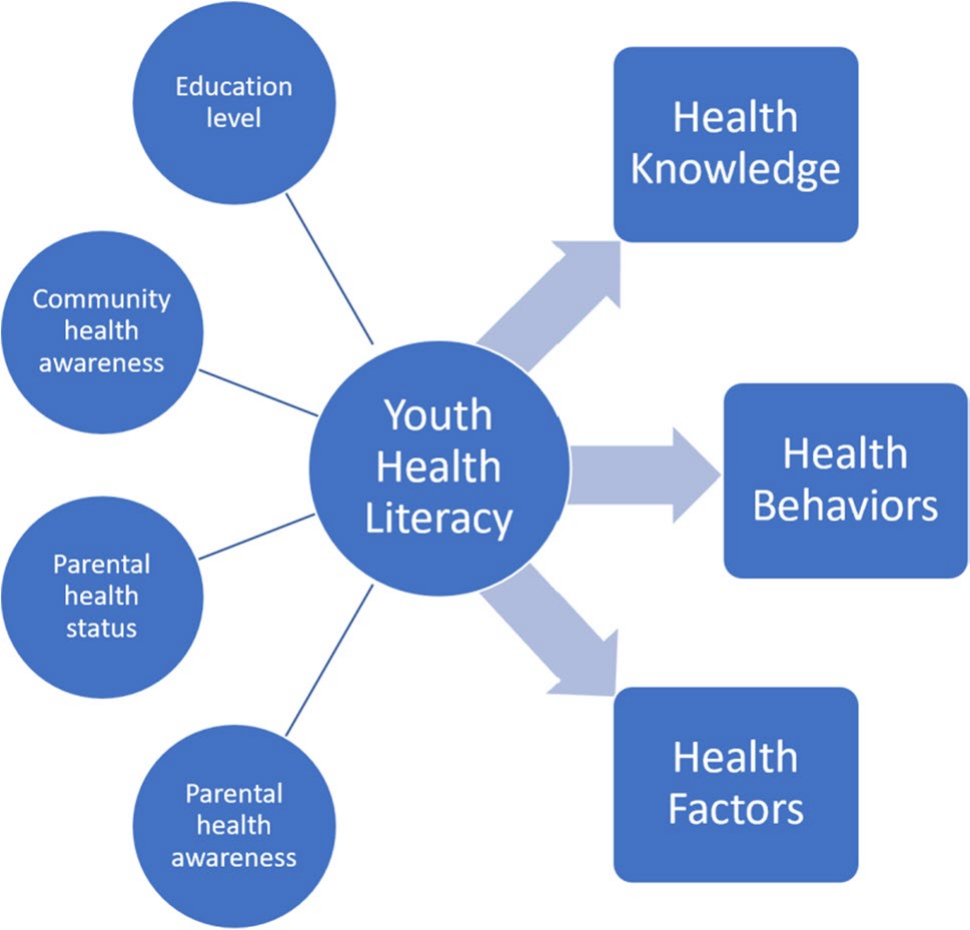
Conceptual framework for youth health literacy

Parental health literacy has a crucial contribution towards health of children and adolescents and is associated with the cardiovascular health of youth. In children with type 1 diabetes mellitus, data have demonstrated an almost 2-point increase in mean hemoglobin A1c percentage in children with caregivers of limited health literacy (10.4 ± 2.2% vs 8.6 ± 1.7%; *p* < 0.001) [35]. Family-based conversation about ASCVD is associated with improvements in health-related knowledge among children and adults [36]. Limited parental health literacy is associated with increased ASCVD risk factor prevalence in youth, such as increased rates of adolescent obesity [37]. Health literacy of both caregivers and youth are instrumental in ASCVD prevention, as outlined in the proposed mechanism in Fig. 1, and should be targeted in future education-based interventions.

Educational programs for youth which include family members have shown stronger lifestyle changes in both caregivers and youth [38]. For example, in a study from China of 603 5th graders, a 3.5-month school-based salt-reduction education program was conducted [39]. The aim of this study was to examine the educational intervention’s effect on the adolescents’ salt-related behaviors, as well as examine associations between the adolescents’ salt-related behaviors and behaviors of those within their social network, including their family members, teachers, and peers. Salt-related behaviors were measured by a survey validated by 24-urine collection in 135 of the participants. The survey asked questions regarding frequency of eating salty snacks and whether participants’ families were incorporating salt reduction practices in their lifestyles, with a max score of 9 indicating the highest-number of salt-reduction behaviors.

Results collected 9 months after completion of the educational program demonstrated that adolescents from families less supportive of salt reduction had lower salt-reduction behavior scores (− 1.1, 95% CI − 1.6 to − 0.6; *p* < 0.0001) [39]. The approach of family integration may apply to the broader unit of community as well. Youth at schools with teachers and principals who received additional education and training about salt-reduction also had higher salt-reduction behaviors, in comparison to youth at schools whose teachers and principals did not participate in additional training (+ 0.33; 95% CI 0.01 to 0.64; *p* = 0.043) [39]. In an associated study conducting the same intervention among 832 youth and 553 family members, family members who received educational programming regarding salt intake and hypertension at the same time as adolescents had a 2.9 g decreased salt intake as calculated by 24-h urinary sodium in comparison to families in the control group who received a standard school curriculum (− 2.9, CI − 3.7 to − 2.2; *p* < 0.001) [40]. These studies suggests that the optimal educational intervention to bolster ASCVD prevention in youth focuses not only on the health literacy of the child, but of the parent and their surrounding community too.

## Conclusion

Health literacy contributes to cardiovascular risk in children and adolescents. Programs that provide accessible education with appropriate tailoring to participants’ level of education have demonstrated success in increasing ASCVD knowledge, healthy behaviors, and healthy habits. Further adding to the known associations of sociodemographic factors such as health literacy and other SDOH, the available data suggest that caregiver and community involvement in educational interventions can enhance the interventions’ effects on children and adolescent’s lifestyle behaviors.
